# Supplementary material for: “I wanna live and not think about the future” what place for advance care planning for people living with severe multiple sclerosis and their families? A qualitative study
Source: PLoS One. 2022 May 26;17(5):e0265861. doi: 10.1371/journal.pone.0265861 (PMC9135191; doi:10.1371/journal.pone.0265861)
Supplement: S1 Appendix — (DOCX) [file pone.0265861.s001.docx]

**S1 APPENDIX: Topic Guide for Person with MS**

***During this interview I’d like to ask you some questions and for you to answer in your OWN words.***

***There are no RIGHT or WRONG answer to ANY of my questions.***

***Please be aware that we can STOP this interview at any time you wish. YOU DO NOT HAVE TO GIVE A REASON. Are you ready to start?***

**Introduction questions**

- I'd like you to think back to when you first became unwell. Please tell me in your own words what happened.
- Thinking back to when you first saw a healthcare professional about your MS, could you please tell me what you remember they said about your illness?
- Do you have views about what you were told? Would you have liked it to be different? ***If so, in what way?***
- How long have been living with MS? In your own words, how do you feel now?
- Has your MS changed how you view yourself as a person? If yes, tell me in what ways?
- Could you please tell me how your care is planned at the moment? Do you feel you have a say in it?

**Quality of life questions**

Medicine has shifted quite a lot in recent years. It's not just simply about controlling physical symptoms people experience as a result of their illness. It's also about trying to make it possible for people to realise their quality of life. I’d now like to talk to you more about some of these issues in relation to your illness and how you feel.

- ‘Quality of life’ is different for everyone. Thinking about the words ‘quality of life’, what do they mean to you? What brings you quality of life? Has this changed over time?

**Considering the future questions**

- Let's look forward to an unknown future about your illness. In what ways do you think about your illness like this? Do you talk about the future with other people? (e.g. family or friends or healthcare professionals caring for you) What sort of conversations do you have?
- Have you ever thought about what the main goal or purpose should be for your medical care? What do you think it should be?
- People’s goals may change at different times in their life. Have your goals changed over time? What do you think they ***might*** look like as your illness progresses?

**Questions about continuing treatment even during very advanced illness**

Now the next questions might potentially be a little more difficult for some people to answer. They focus more on the care at ***or near*** the end of life for some people who are living with life-limiting illness, and their families. Please do not hesitate to let me know if you would rather we do not go on any further. If at any point you would rather we stop let me know.

- Have you ever known anyone with the same illness as you, or perhaps with another illness like cancer, that have had to make important decisions about their future care? What do you think was most important to them? Now thinking about your own situation what would be most important to you and why?

*(Possible prompts might include: thinking about living for as long as possible even if the participant has a poor quality of life. Or, to try treatments for a period of time, but stop if the participant is suffering. Or to focus on the quality of life and comfort, even if the participants’ life is shorter)*

**Questions about advance care planning**

- I would like to ask you if any of the healthcare professionals you see have ever used the words **‘advance care planning’** with you. If you have, please tell me what they mean to you?

**Please take a few moments to think about the two statements on this laminated card. I will then want to ask you some questions in relation to them.**

**SHOW CARD**

**Advance care planning is a process that supports adults at any age or stage of health in understanding and sharing their personal values, life goals, and preferences regarding their future medical care.**

**The goal of advance care planning is to help ensure that people receive medical care that follows their values, goals, and preferences during serious and chronic illness. For many people, this may include choosing and then informing another person or people they really trust to make medical decisions in the event they can no longer make their own decision.**

- Could you please tell me in your own words what this first statement means to you?
- In what ways do you think ‘advance care planning’ would be useful to people living with MS and their families?
- Have you ever talked to someone about what you would want to happen if you could no longer make decisions for yourself? Have you recorded your wishes in writing?

**If ‘yes’ to the above ask the following questions**

- When did you think it was the right time to plan and write down what you wanted?
- Did you see the process of thinking about and then recording your wishes as an activity that just involved you? Why was this the case? Alternatively, you might have involved others in the discussions and decisions you made. If this is the case, who did you discuss these issues with and why?
- What sort of issues did you discuss and then write down?
- ***What would a ‘successful’ advance care plan look like (or consist of) for you?***
- Where did you talk about these important issues?
- Have you changed your mind about what you wrote at any point?

**If ‘no’ to the above ask the following questions**

- When do you think it would be appropriate to talk about planning for the future?
- Do you see the process of thinking about and then recording your wishes as an activity that might just involve you? Why would this be the case? Alternatively, you might want to involve others in the discussions and decisions you make. If this is the case, who did you would you discuss these issues with?
- Who would you want to talk to about these important issues?
- ***What do you think a ‘successful’ advance care plan would look like or consist of?***
- Where would you want to talk to about these important issues?
- How often do you think it would be reasonable to revisit the decisions you make about your future care?

**The COVID-19 pandemic**

I’d like to ask you to reflect on COVID-19, a situation that has changed so many people’s lives and how they feel about many issues.

- Has COVID-19 changed the way you feel about your illness and your situation? ***If so, in what ways?***
- Has COVID-19 changed your views about the advance care planning? ***If so, in what ways?***
- Have any health professionals discussed your MS in relation to COVID-19? ***If so, in what was discussed? How did you feel as a result of the conversation?***

**Concluding questions**

- I'd like to end this interview by asking you in your own words what makes you happy. How has what makes you happy changed as your illness has progressed?
- Finally, are any other issues you would like to talk about with me, either about the topics we've discussed today or about the study?
