# Supplementary material for: “I wanna live and not think about the future” what place for advance care planning for people living with severe multiple sclerosis and their families? A qualitative study
Source: PLoS One. 2022 May 26;17(5):e0265861. doi: 10.1371/journal.pone.0265861 (PMC9135191; doi:10.1371/journal.pone.0265861)
Supplement: S2 Appendix — (DOCX) [file pone.0265861.s002.docx]

**S2 Appendix**

Ethical Discussion Group (EDG)

Topic Guide

| **Research objectives for ethical discussion group (EDG):**   - To explore health and social care professionals’ views on the content, place, timing and challenges involved in discussing advance care planning (ACP) among people with multiple sclerosis (PwMS), and their families - To invite group participants to critically reflect upon the ideas and issues presented to them from Workstreams 1 (literature review) and 2 (interviews with PwMS and family members) (to be shared in document format in advance of the EDG) - To examine group participants’ reasoning and justification for beliefs, practices and ethical principles underlying their negotiation of ACP - To highlight the challenges when engaging PwMS and their families in this process - To identify potential solutions that can inform the development of workable and accessible guidance on ACP |
| --- |

**Note on use of EDG topic guide:**

*The following guide does not contain pre-set questions but rather lists potential key themes and sub-themes to be explored within the EDG. We wish to encourage participants to discuss their views and experiences in an open way without excluding unforeseen issues that may be of importance to participants and the study as a whole. The moderator (Professor Bobbie Farsides, Professor of Clinical and Biomedical Ethics at Brighton and Sussex Medical School) will use the guide to formulate questions which are responsive to the group discussion as it evolves. Bobbie Farsides has extensive experience using this methodology and over a twenty-year period has used EDGs to help identify the complex ethical, legal and clinical issues that healthcare professionals and medical scientists experience when working in a range of areas, including assisted reproductive services, embryology, stem-cell research, and solid organ donation. The order in which topics are explored will be flexible, according to the priorities that emerge within the group. Differing and shared views, experiences and attitudes will be explored throughout in order to understand how and why views, behaviours and experiences have arisen.*

1. Welcome & introductions/housekeeping

- Introductions: moderator and research team
- Introductions: group participants (ask participants to introduce themselves, stating their name, job title, and to briefly outline their involvement with people severely affected by MS and their families)
- Introduction to research: funded by the MS Society to develop ACP resources for people severely affected by MS and their families
- Reason for asking them to participate
- Brief introductions from group participants
- Remind re consent process, confidentiality and anonymity, use of digital recorder and data storage
- Length of group discussion (1.5 hours) and nature of discussion (specific topics to cover but will be like a conversation, ‘there are no right or wrong answers, in your own words’)
- Important to hear from everyone in the group and that participants do not talk over each other
- Remind that group discussion will cover issues that may be perceived by participants as being sensitive and potentially distressing. Participants are free to decline to contribute to any aspects of the discussion without giving a reason
- Zoom/Microsoft Teams etiquette
- Role of moderator in guiding the discussion
- Role of research team (as observers only)
- Any questions?

1. Views on utility of ACP for PwMS and their families
   1. Objectives of ACP for PwMS and their families
   2. Perceived usefulness / advantages / benefits
   3. Perceived disadvantages
   4. Importance of involvement of healthcare professionals (HCPs) in ACP for PWMS and their families
2. Views on content of ACP for PwMS and their families
   1. What ACP should include/involve

E.g.

- - 1. Identifying values and preferences for care and treatment in the event of decisional incapacity (e.g. based on past experiences and quality of life)
    2. Choosing proxy/surrogate decision makers
    3. Choosing whether to grant leeway in proxy decision making
    4. Shared understanding between patients, proxy/surrogate decision makers and wider family, and HCPs
    5. Value of different ACP documents
       1. Legal (Lasting Power of Attorney, Advance Decision to Refuse Treatment, Do Not Attempt Cardiopulmonary Resuscitation order)
       2. Non-legal (advance statement of values and preferences)

1. Views on timing of initiating ACP discussions with PwMS and their families
   1. When ACP should start (e.g. triggers for initiating ACP)
      1. Transition points (e.g. end of active treatment, secondary progressive diagnosis)
      2. Unplanned acute hospital admission for complications associated with MS
      3. Progressive loss of cognitive capacity
      4. Progressive loss of physical capacity (e.g. swallowing)
   2. ACP as a ‘one-time’ event or a process
2. Views on who should initiate ACP, and who should be involved, where
   1. Initiation of ACP by HCPs
      1. Neurologist?
      2. MS Nurse?
      3. GP?
      4. Other?
   2. Involvement of families
   3. Where might ACP be initiated (setting)
3. Views on challenges to initiating ACP with PwMS and their families
   1. Challenges for PwMS and their families
      1. Uncertainty and acceptance/non-acceptance of progressive nature of illness
      2. Positive or negative view of ACP
      3. Barriers within families to discussing ACP
      4. Depression in MS and ACP
      5. Confusion and memory loss and ACP
   2. Challenges for HCPs
      1. Skills/training needs (talking about the future/balancing hope and realism)
      2. Strength and duration of relationship with PwMS and their family
      3. Confidence
      4. Time/resources
      5. Processes and how to incorporate into practice
      6. Whether endorsed/required as part of care
4. Identifying potential solutions to challenges
   1. Information and guidance for PwMS and families
      1. Format(s)
      2. How accessed, where from
   2. Information, guidance, training for HCPs
